# Positive Adolescent Development: Effects of a Psychosocial Intervention Program in a Rural Setting

**DOI:** 10.3390/ijerph17186784

**Published:** 2020-09-17

**Authors:** Diana Paricio, Maria F. Rodrigo, Paz Viguer, Marina Herrera

**Affiliations:** 1 Department of Psychology and Education Sciences, Open University of Catalonia, 46003 Valencia, Spain; 2 Department of Methodology for the Behavioural Sciences, University of Valencia, 46010 Valencia, Spain; maria.f.rodrigo@uv.es; 3 Department of Developmental and Educational Psychology, University of Valencia, 46010 Valencia, Spain; 4 Department of Social Psychology, University of Valencia, 46010 Valencia, Spain; marina.herrera@uv.es

**Keywords:** adolescents, positive development, intervention program, rural context

## Abstract

The Positive Youth Development (PYD) approach identifies adolescents as resources to be empowered rather than problems to be solved. All adolescents have strengths and will fully develop when these strengths are integrated with healthy resources in the diverse environments where they live and interact. The objective of this study was twofold: (1) to present the Positive Development Program for Adolescents living in rural areas (DPAR Program) and (2) to pilot test the intervention program. The DPAR program was evaluated using a repeated-measures design before and after the intervention, with an intervention group and a control group. The sample consisted of 176 adolescents between 11 and 15 years old (M = 12.89, SD = 0.90) who belonged to two high schools with similar characteristics located in rural settings. A mixed-design analysis of variance was performed for each dependent variable. Results showed a significant increase in most of the study variables (self-esteem, self-efficacy, group identity, empathy, relational skills, assertiveness, and conflict resolution) and a significant decrease in alexithymia, as well as better academic performance. All this evidence indicates that the DPAR program is effective in promoting positive adolescent development and addresses the lack of programs based on the PYD approach in rural areas.

## 1. Introduction

In recent decades, models that focus on promoting competencies in adolescence have proliferated [1,2,3,4] because a reduction in or absence of risk factors does not necessarily ensure healthy development in adolescents [5,6]. These models recognize that the adolescent has great potential for growth [7], and that strengthening his/her assets through relationships and contexts that promote this development fosters the adolescent’s well-being and full development [8,9].

The present study is framed within the Positive Youth Development (PYD) approach, which highlights the importance of the adolescents’ psychological, emotional, and social wellbeing. It is based on the idea that adolescents have strengths that can be developed, rather than being the bearers of problems to solve [6,7]. From this point of view, interventions based on universal social and emotional learning (SEL) can be considered the best ones for predicting adolescents’ long-term wellbeing [10].

Today, although the philosophy and objectives of PYD are clearly articulated [6,7], the main components of an effective PYD program are not clear. For Brooks-Gunn and Roth [11], one of the greatest challenges in studying PYD is how to conceptualize and measure contexts. These authors believe that, without a doubt, research prior to the PYD approach studied the diverse contexts in which young people find themselves. There was a solid body of literature on families and communities, as well as on peer groups, both in schools and in communities. However, the focus was generally on how these contexts were associated with the emergence of negative behaviors, rather than positive.

Intervention with adolescents requires understanding the factors associated with adolescent risk and the ways they acquire and master the skills needed to promote healthy development. Waid and Uhrich [12] recently conducted a broad review of the theory and practice of PYD in which they reviewed 65 articles published between 1997 and 2017. The results suggest that PYD programs are diverse and can be adapted to a variety of adolescent needs across geographical and cultural contexts; however, due to methodological heterogeneity, empirical support is not the same for all the programs. Also, Ciocanel et al. [13] recently conducted a meta-analysis of 24 PYD interventions in which they analyzed the impact of interventions on behavior problems, risky sexual behavior, academic performance, prosocial behavior, and psychological adjustment. The interventions had a small but significant effect on academic performance and psychological adjustment. No significant effects on risky sexual behavior, problem behavior, or positive social behaviors were found.

The three main PYD models are: The Five Cs model [6], the Developmental Assets model [7], and the Positive Adolescent Development model [3]. The predominant model in PYD is the Five Cs model by Lerner et al. [6]. This model consists of: Competence (ability to do something successfully in academic, work, and social settings); Confidence (overall positive self-esteem and self-efficacy); Connection (positive and strong relationships with peers, family, school, and community); Character (respect for social and cultural norms, acquisition of appropriate role models, sense of right and wrong, and integrity); and Caring (feelings of sympathy and empathy and identification with others). Finally, when the adolescent exhibits behaviors that are consistent with the five Cs, a sixth C, Contribution, emerges (positive behaviors toward self, family, community, and society). This theoretical model has been empirically validated through a longitudinal study in the USA with a large sample of adolescents and young [2,14]. Regarding the contextual assets, that is, factors promoting PYD in any context, the Developmental Assets model by Benson et al. [7] has received a high level of consensus in the USA. Based on the review of empirical evidence on resilience, the authors propose a series of contextual assets (strengths present in the community) and internal assets (skills, competencies, and personal commitments) for the promotion of well-being and healthy adolescent development [7]. These assets are divided into eight main dimensions: (1) supports, (2) empowerment, (3) limitations and expectations, (4) constructive use of time (external assets), (5) commitment to learning and school, (6) positive social values, (7) special competencies, and (8) positive identity (internal assets).

In addition, Oliva et al. [3] developed a theoretical model that includes the most important competencies for adolescents’ development. Specifically, this model defines healthy and positive youth development, based on 27 specific competencies grouped in five main areas: personal, social, cognitive (including academic competence), emotional, and moral development. Personal competence holds a central position, whereas the others are interrelated and together strengthen personal identity. These are basic competencies, capabilities, and abilities that support the other competencies and, in turn, help to develop them.

The three models have similar characteristics, and they all contain important positive youth development variables such as self-esteem, assertiveness, self-efficacy, and empathy (as Table 1 shows). However, Oliva’s model differs from the other two because some moral, emotional, and cognitive competencies are described more and grouped differently [3]. All the competencies included in the model can be considered quantitative dimensions or a continuum on which adolescents will be placed according to their mastery of each. Therefore, it is not a question of having or not having a competence, but rather of showing a certain degree of mastery of it. Although they are not expected to achieve very high values on many of these competencies in early or middle adolescence, these are goals or skills that must be promoted in order to foster positive and healthy development during these years. Another difference with regard to the other two models is that after the model was created by a large number of experts and professionals in the field of adolescence, it was validated by a large sample of Spanish adolescents through a quantitative study. The results of the factorial analyses carried out on the competencies or variables evaluated explained 86.69% of the variance, with a higher percentage of variance in the personal competencies, which justifies their central position in the model.

Empirical studies have shown that interventions based on a positive developmental approach maintain the benefits they produce in adolescents in the long term [10,15]. Moreover, by significantly improving skills, positive attitudes, prosocial behavior, and academic performance, they also serve as a protective factor against the emergence of later problems in the form of criminal behavior or clinical disorders [3,5,10,16,17,18].

At the international level, we find some programs based on the 5C model: the 4-H youth programs with wide state coverage in the USA [2,6], Positive Adolescent Training through Holistic Social Programs (Project PATHS) in Hong Kong [19], outdoor education programs based on PYD in New Zealand [20], or the Changing Lives Program [21]. In Spain, few programs have been designed specifically to stimulate PYD, and even fewer proposals have been evaluated and demonstrated their effectiveness [3]. Some examples are the Personal and Social Responsibility Program [22], the Program of activities in nature to promote personal and social responsibility in vocational education students [23], or the psycho-educational and community intervention program Building My Future [24], designed for adolescents between 11 and 18 years old. The program results show significant changes in self-concept, empathy, problem-solving strategies, and feelings of being integrated in the community in groups of adolescents at psychosocial risk [25].

Based on the model of positive adolescent development by Oliva et al. [3], the aim of this study is to implement a psychosocial intervention pilot program in the school environment in a rural setting and evaluate its effects.

### Positive Development Program for Adolescents Living in Rural Settings (DPAR)

The DPAR Program takes into account four significant elements in positive adolescent development: family, school, peers, and community. In this regard, it differs from previous PYD programs, such as extracurricular programs with community participation [14,20,23,24] or school programs with involvement of teachers or external staff [19,22]. In Spain, there are few programs in which young adults from the community participate in classrooms, the ideal place to promote PYD, as referents to promote positive behaviors, and this is especially true in the rural environment. However, it is important to keep in mind that the school is the most common meeting place in rural areas [26].

Based on the PYD model by Oliva et al. [3], the Five Cs model [6], and the Developmental Assets model [7], the specific objectives of the DPAR Program were: (1) To promote personal development: self-esteem, self-concept, and self-efficacy; (2) To develop the group identity of the class and ties among peers; (3) To improve interpersonal communication skills, both among peers and with adults: verbal and non-verbal communication, assertiveness, praise, giving and receiving complaints, and resisting group pressure; (4) To learn effective conflict resolution strategies and coping mechanisms; (5) To develop critical and analytical thinking in certain situations of daily life: ability to plan and make decisions. (6) To encourage prosocial behavior and the acquisition of values: sense of justice, respect for diversity, equality, and social responsibility; (7) To enhance the skills of knowing and managing one’s own and other people’s emotions, empathic capacity, and tolerance to frustration; (8) To improve academic performance.

In accordance with the specific objectives, the variables selected to evaluate the DPAR Program were the following: self-esteem, self-efficacy, and identification with the class were included as variables in the personal area (see Table 1). Self-esteem can be considered one of the most powerful predictors of the degree of psychological adjustment during adolescence [27,28]. Self-efficacy is fundamental in the school setting because it affects not only academic performance, but also quality of life in relation to adolescent health [29,30], and it is also a good predictor of satisfaction with life [29,30]. Identification with the class favors students’ well-being and commitment [26,31,32].

The social area includes the variables of relational skills, assertiveness, and conflict resolution skills. Social skills training is based on the importance of interpersonal relationships for good psychological adjustment [16,33,34], in addition to encouraging positive leadership behaviors in the community [3,35].

The emotional area includes empathy and alexithymia. Empathy and emotional skills are strongly related, and they make it possible to acquire and keep friendships and manage social problems more effectively [36]. Empathy is a predictor of personal and social responsibility [37,38], and empathic adolescents are less aggressive [39]. In addition, emotionally intelligent adolescents have greater physical and psychological health and know how to better manage their emotional problems [40].

The cognitive area includes the planning and decision-making variable. This complex skill is developed throughout adolescence and should be fomented in order to compensate for the imbalance that exists between cognitive and motivational nerve connections that produce a certain vulnerability and increase impulsiveness and risk-taking [34,41]. Responsible and autonomous decision making leads the adolescent to achieve good psychosocial adjustment and greater social commitment [42].

The moral area includes moral values. Learning and integrating positive values significantly improves adolescents’ attitudes towards their environment and the development of prosocial behavior [3,10,17,18].

Finally, academic performance has been included as a cognitive competence. In educational contexts, there has been constant interest in understanding the cognitive and behavioral factors that favor or hinder students’ performance on their academic tasks and the way these factors are related to their overall development [43,44]. Moreover, empirical evidence highlights the connection between academic performance and the development of social-emotional competencies [45,46].

The aim of the study was to pilot test the intervention. The program aims to target all the above variables in order to see whether they improve after the intervention. In this sense, the main objective of the present study was to carry out a pilot test of the psychosocial interventionThe hypotheses proposed are that, after implementing the program, the intervention group will obtain higher scores than the control group on self-esteem, self-efficacy, group identification, empathy, relational skills, assertiveness, conflict resolution, planning and decision-making, moral values, and academic performance; and lower scores on alexithymia.

## 2. Materials and Methods

### 2.1. Participants and Study Design

To evaluate the effects of the DPAR program, a repeated-measures (pre-test and post-test) quasi-experimental design was used with an intervention and a control group. The sample is composed of 176 students (50.6% girls and 49.4% boys) between 11 and 15 years old ($\overset{-}{x}$ = 12.89, SD = 0.897). The participants belonged to two high schools (compulsory secondary education) located in a rural setting (Intervention Group = 83; Control Group = 93). Of them, 45.5% were in 7th grade, and 54.4% were in 8th grade. Furthermore, 71.3% of the sample has never repeated a school year, whereas 24.7% have repeated at least one course.

The two high schools are in a rural setting. The intervention group belongs to the Sierra de Gúdar-Javalambre in the province of Teruel, and the control group belongs to the Mancomunidad de la Canal de Navarrés in the province of Valencia, both in Spain.Students come from different municipalities, 7 to 14 municipalities (municipalities with low population density, an elderly population, with shortages in public transport and limited leisure alternatives for adolescents). These high schools generally have a large number of interim teachers, which limits the possibility of carrying out consistent projects over time. The work profile of the families is as follows: father with low-medium-skilled work, and 40% of mothers are housewives. The cultural context is characterized by low reading habits and little participation in associations; in addition, the opportunities are quite limited.

To find out whether the intervention and control groups show differences on sociodemographic variables at pre-test, homogeneity analyses were performed. Tests for homogeneity revealed no significant differences between groups at pre-test on the variables: age, sex, year in school, and academic marks (see Table 2).

### 2.2. Procedure and Structure of the Intervention Program

Various informative meetings were held with the selected schools to explain the objectives and methodology of the DPAR program in June 2016. The high schools were selected through non-probability convenience sampling based on their accessibility and previous interest in participating in this study. After obtaining parent permission and authorization, one school was assigned to the intervention group and participated in the DPAR program in the 2016/2017 school year, and another school was assigned to the control group and did not participate in the program. The instruments were administered in the students’ usual classroom by a professional who was not associated with the school, for a period of 45 min. The students were informed that their participation in the study was voluntary, anonymous, and confidential. None of the students refused to participate.

With regard to the program’s structure, it consists of 21 units integrated into fivemodules: personal, emotional, social, cognitive, and moral competencies, in addition to introductory and closing modules (see Table 3). All the sessions are held weekly during school hours. Each unit requires one or two sessions that last approximately 50 min, with the weekly participation of the teachers and a former student of the school, as well as the family’s participation indirectly through homework. Therefore, in the classroom, the professional specialized in PYD, the teacher-tutor, and a former student of the school are present. In the first part of the session, the former student is introduced (10–15 min.). Next, the competence or skill proposed for that week is developed (30 min.). Finally, the homework from the previous session is addressed, and/or the task the adolescents have to work on with their families is explained (10 min.). The activities carried out include debates, work groups, dramatization, role-playing, etc. Both current and future situations close to the adolescent’s context are presented.

The intervention is structured in three phases:

*Phase 1. Preparation*. This is a very important phase in the program because the preparation tasks make it possible to raise awareness and prepare the scenario for the practical incorporation of personal, cognitive, social, moral, and emotional competencies in the classroom. Previous tasks of contact, awareness, and program preparation are carried out with members of the educational community and former students in a large classroom in the school. This phase lasts one month and makes it possible to reach a consensus on the bases for the DPAR Program, preferably in the month of July, before the end of the school year. The actions carried out have the following two objectives: (a) to establish the philosophy of positive development in the school and the way it is included in the Curriculum Project of the School and the Parents’ Association; and (b) to create community involvement in the promotion of the adolescents’ positive development through the participation of former students of the school. First, time is allotted to meet the teachers and the Guidance Department team through formal and informal meetings about the DPAR Program, the way it is going to be implemented, and what its functions are. Second, a meeting is arranged with the members of the Parents’ Association and the families through a formal call for the presentation of the DPAR Program. Later, once the school year has begun, a letter is sent presenting the program and attaching a parental authorization to participate in it. Third and finally, the necessary material and/or human resources and the way to obtain them are established. In this first phase, the pre-intervention evaluation with standardized scales is also carried out with the students, both the students who participate in the program and a control group in the students’ regular class. This pre-evaluation is performed in one of the initial sessions of the program.

*Phase 2. Intervention*. In this phase, the intervention tasks are carried out with students, teachers, and former students of the school. It lasts eight months and allows the development of personal, social, cognitive, moral, and emotional skills directly in the students who participate, and indirectly in the teachers, family, and community (specific activities are shown in Table 3).

The students, who are the main target group, have practical sessions. These sessions are held during school hours, and in them, the adolescents learn skills that will allow them to fully develop in the present moment and in adulthood.

The teachers, the secondary target group, carry out formative and experiential workshops on positive development, and they participate in the students’ sessions. On the one hand, in the training workshops, theoretical training is provided that includes: (a) Knowing about the negative view and pathologizing of adolescence; (b) Theoretically, knowing about the models of positive development, from the deficit model to the current models of positive development; and (c) Learning the intervention techniques used in the DPAR Program to participate in the sessions. To do this, the following process is followed: (a) The constructs of positive development are reviewed; (b) The intervention techniques to favor their development are presented; and (c) The content and development of the workshops with the students are learned in order to participate in the intervention sessions. On the other hand, the strengths and weaknesses of the school are evaluated in order to propose strategies that promote a warm and safe social climate for the adolescents’ greater well-being and psychological adjustment. In this regard, the aim is for the teachers to have good social-emotional skills and be able to relate to others and solve the problems that arise in the classroom and, in general, focus their work positively.

With the family, also a secondary target, an informative and formative session is held before the end of the course that precedes the beginning of the implementation of the DPAR Program. Later, a dossier is sent with the contents, activities, and students on a quarterly basis to encourage them to participate in their children’s teaching and learning process. In addition, in a transversal way, various tasks the students have to perform at home necessarily involve the parents.

The community refers to the former students from the different municipalities who have studied at the same school, reside in that context, and/or maintain some type of link with their town (emotional, family, work, etc.).

A training workshop is held with all the participants on the philosophy of the DPAR Program and the structure of each session. Afterwards, a training session is held with each former student to develop the competence addressed on the day they are participating and the methodological indications to transmit their life project to the students.

At the halfway point of the intervention, the ad hoc satisfaction questionnaire is administered to all the students anonymously to assess their satisfaction with the intervention, the aspects they would like to address in greater depth, and the skills they would like to see promoted.

*Phase 3. Closure and feedback on the program*. In June, the students complete the post-intervention measures (post-test). The collection, analysis, and presentation of the results of the program to the entire educational community is carried out to constructively close the program.

### 2.3. Measures

*Self-esteem***.** To assess self-esteem, the Spanish adaptation by Echeburúa [47] of the Rosenberg Self-Esteem scale (RSE, [48]) was used. It is composed of 10 items (for example, “I think I have a lot of reasons to feel proud”) rated on a Likert-type scale with response options ranging from 1 (strongly disagree) to 4 (strongly agree). Cronbach’s alphas were 0.70 (pre-test) and 0.76 (post-test).

*Self-efficacy*. The General Self-Efficacy Scale [49], validated in Spain by Sanjuán et al. [50], was used. It is a unidimensional scale composed of 10 Likert-type items (for example, “Thanks to my qualities and resources, I can overcome unexpected situations”), where 1 represents *strongly disagree* and 4 represents *strongly agree*. Cronbach’s alphas were 0.81 (pre-test) and 0.89 (post-test).

*Group Identification*. Tarrant’s Group Identification Scale [51], adapted in Spain by Cava et al. [52], was used. The scale has 13 items (for example, “I am happy to belong to this class”), with a Likert type scale (0 = strongly disagree, 10 = strongly agree). The students were instructed to respond to the questionnaire by considering the class as the group. Cronbach’s alphas were 0.81 (pre-test) and 0.88 (post-test).

*Empathy*. To measure this variable, the Basic Empathy Scale by Jolliffe and Farrington [53], adapted in Spain by Oliva et al. [34], was used. The adapted scale has nineitems (for example, “Other people’s feelings affect my happiness”), rated on a Likert-type scale (*1 =* strongly disagree*, 5 =* strongly agree). Cronbach’s alphas were 0.75 (pre-test) and 0.79 (post-test).

*Alexithymia*. To assess this variable, the Toronto Alexithymia Scale (TAS-20) by Bagby, Taylor, and Parker [54], Spanish adaptation by Sánchez-Sosa [55], was used. It has 20 items (for example, “It is difficult for me to find the right words to express my feelings”) rated on a Likert-type response scale (1 = strongly disagree; 6 = strongly agree). Cronbach’s alphas were 0.82 (pre-test) and 0.79 (post-test).

*Social Skills*. Scale of Social Skills of Olive et al. was used [34]. It has 12 Likert type response items ranging from 1 (Totally false) to 7 (Totally true). The three subscales were used: Relational Skills, Assertiveness, and Conflict Resolution. The reliability coefficients, Cronbach’s alphas, of the subscales for Time 1 were 0.74, 0.68, and 0.65, respectively, and for Time 2, 0.79, 0.70, and 0.74, respectively.

*Planning and Decision Making*. The Problem Solving/Decision Making Subscale of the Life Skills Development Scale for Adolescents by Darden et al. [56], adapted by Oliva et al. [34], was used. It has eightitems, ranging from 1, strongly disagree, to 7, strongly agree). Cronbach’s alphas were 0.84 (pre-test) and 0.87 (post-test).

*Moral Values*. The Scale of Values for Positive Adolescent Development by Oliva et al. [34] was used. It has 24 items, with a Likert-type response scale (1 =Not at all important; 7 =The most important thing). Cronbach’s alphas were 0.90 (pre-test) and 0.88 (post-test).

*Academic Performance*. To evaluate this variable, the average of the student’s grades in the first evaluation (in December) and at the end of the course (in June) was used. These grades are grouped into fivecategories, rated on a scale from 0 to 10 (1 = Insufficient; 10 = Excellent).

### 2.4. Statistical Analysis

The Statistical Package for the Social Sciences (SPSS) version 25.0 for Windows (IBM Corp., Armonk, NY, USA), was used for data analysis. The comparability of the intervention and control groups on the pre-test was assessed using independent t tests or chi-square tests. To evaluate the effects of the program on each of the study variables, several 2 × 2 mixed-design analyses of variance (ANOVA) models were conducted, with a between-subjects factor (intervention or control group) and a within-subjects factor (pre-test and post-test).

This is the recommended analysis when working with non-random or pre-existing groups [57], as in the case when schools are assigned to conditions [58].The eta-square partial ($n_{p}^{2}$) value is used as an indicator of the effect size. Cohen [59] suggested that $n_{p}^{2}$ ≤ 0.06 can be considered a ‘small’ effect size, 0.07 ≤ $n_{p}^{2}$ ≤ 0.14 represents a ‘medium’ effect size, and > 0.14 is a ‘large’ effect size. Simple effects analyses were conducted after statistically significant interaction effects in ANOVAs to assess the significance of the change from pre-test to post-test in the intervention and control groups.

## 3. Results

Analyses were performed to test for possible differences in pre-test means (Table 4) between the intervention and control groups on PYD variables. The mean comparisons were statistically significant for alexithymia (*t* = –2.27, *p* = 0.024), conflict resolution (*t* = 2.95, *p* = 0.004), and planning and decision making (*t* = 3.76, *p* = 0.001).

**Figure 1:**
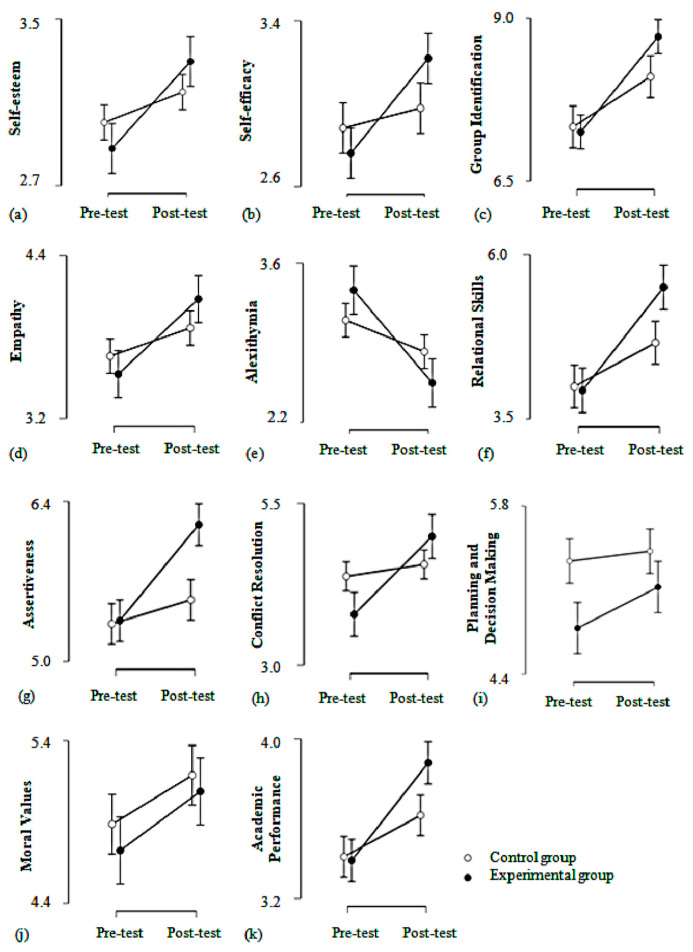
Pre-test and post-test means on PYD variables for intervention and control groups. (**a**) Self-esteem, (**b**) self-efficacy, (**c**) group identification, (**d**) empathy, (**e**) alexithymia, (**f**) relational skills, (**g**) assertiveness, (**h**) conflict resolution, (**i**) planning and decision making, (**j**) moral values, (**k**) academic performance.

Mixed-design group (intervention/control) × time (pre-test/post-test) ANOVA models were performed on each of the PYD and academic grade variables, with group as between-subject factor and time as within-subject factor. In each case, we mainly focused on the group-time interaction effect, which would indicate a differential change between pre-test and post-test for the two groups. The descriptive statistics and ANOVA results are presented in Table 4. The plots for the pre-test and post-test means in the intervention and control groups are presented in Figure 1.

ANOVA results showed significant group-time interaction effects for the following variables: self-esteem (*F* (1, 153) = 6.79; *p* = 0.010; $n_{p}^{2}$ = 0.04), self-efficacy (*F* (1, 169) = 8.71; *p* = 0.004; $n_{p}^{2}$ = 0.05), group identification (*F* (1, 167) = 5.48; *p* = 0.020; $n_{p}^{2}$ = 0.03), empathy (*F* (1, 170) = 5.30; *p* = 0.022; $n_{p}^{2}$ = 0.03), alexithymia (*F* (1, 150) = 9.09; *p* = 0.003; $n_{p}^{2}$ = 0.05), relational skills (*F* (1, 109) = 7.43; *p* = 0.007; $n_{p}^{2}$ = 0.06), assertiveness (*F* (1, 170) = 12.14; *p* = 0.001; $n_{p}^{2}$ = 0.06), conflict resolution (*F* (1, 166) = 12.78; *p ≤* 0.001; $n_{p}^{2}$ = 0.07), and academic performance (*F* (1, 174) = 25.64, *p ≤* 0.001, $n_{p}^{2}$ = 0.128). The effect sizes ($n_{p}^{2}$) were moderate for relational skills, assertiveness, and conflict resolution, large for academic performance, and small for the other dimensions. The interaction effect was not statistically significant for moral values or planning and decision making. For these two variables, the main effect of time was statistically significant, showing a significant change from pre-test to post-test, but with the same magnitude in both the intervention and control groups. Based on van Breukelen’s suggestions [57], an ANCOVA was also performed, treating the pre-test as a covariate, and statistically significant differences were obtained in the post-test means of the control and intervention groups on the same variables for which the interaction effect of the ANOVA was statistically significant.

Examination of simple effects (Table 5) revealed that for the self-esteem and empathy variables, there was a statistically significant increase in the means from pre-test to post-test in the intervention group (*p*< 0.001), and a marginally significant change in the control group (*p* = 0.052 and 0.058, respectively). The effect sizes (Cohen’s d) were larger in the intervention group (*d* = 0.79).

For self-efficacy, relational skills, assertiveness, conflict resolution, and academic performance variables, statistically significant changes were observed in the intervention group (*p*< 0.001), but not in the control group. The effect sizes were medium or large, ranging from 0.52 for academic performance to 0.94 for relational skills.

Finally, for the group identification and alexithymia variables, although the pre-test to post-test change was statistically significant in both the intervention and control groups, the significance of the interaction effect for these variables indicates that the change from pre-test to post-test was significantly greater in the intervention group. For all two variables, the effect size in the intervention group was large (>0.80).

## 4. Discussion

This study presented a pilot test of the DPAR Program (objectives, target group, structure, contents, methodology, and evaluation), carrying out an annual school intervention and evaluating the effects in a sample of early adolescents. The results show significant improvements in the intervention group, compared to the control group, in the variables of self-esteem, self-efficacy, group identification, empathy, relational skills, assertiveness, conflict resolution, alexithymia, and academic performance. The results highlight the need to take the context into account, in this case the rural context, in any psychosocial intervention, and the use of the systemic approach (students, teachers, family, and community) to carry out effective PYD-based interventions.

The DPAR program focuses on promoting adolescent well-being through the development of competencies [3,6] that facilitate better adaptation to the new challenges they have to face [7,11]. The intervention program implemented stimulates overall positive development and represents an advance in this area, given that the few intervention programs developed in Spain have been carried out from the perspective of the deficit model or in only one or two specific areas [3]. A second point to note, as suggested in the literature [60,61], is that the context and culture where the intervention is carried out were taken into account. Thus, we approach the Version 3.0 PYD programs proposed by Roth andBrooks-Gunn [11]. On the one hand, through the participation of young adults who are knowledgeable about the school and the rural setting, adolescents are exposed to models that allow them to visualize different life projects and establish positive, close, and trusting relationships, which are considered essential for adolescent development [5,62,63]. On the other hand, a higher educational level of the adults fosters a greater predisposition to reach a high level of studies in young people, thus reducing school dropout [64]. This situation is scarce in rural settings, and so it was considered appropriate to create this link with the participating adults as a resource to stimulate continuity in the adolescents’ training.

Third, following the recommendations of previous studies [5,11,16], the implementation was structured and consistent over time, with three educators per classroom (psychologist, tutor, and alumnus), and using a methodology that promotes personal growth. A wide variety of contents are introduced, as well as positive, close, and supportive interactions of significant others, such as between teachers and peers [65] or between alumni and students of the same high school. All of this created a favorable learning environment [5,66] with a highly motivating component because these activities differ from the strictly curricular activities usually carried out in the classroom. In addition, most of the activities took place in the classroom, which contributed to fostering a sense of belonging and identification with the class [26]. Studies have shown that a fundamental aspect that can favor the development of identification with a group involves carrying out programs that include activities performed with the whole group [67].

Finally, several authors highlight the need to improve the methodology used to evaluate programs that promote competencies in adolescents [5,68]. Therefore, methodological rigor was guaranteed in the evaluation of the program’s effectiveness. Measurement instruments with demonstrated reliability and validity were used, and a control group was included, with pre-test and post-test measures in both groups.

This study is consistent with the existing literature on the effectiveness of youth programs. The DPAR program has shown positive results that support and extend those obtained through other programs with different personal competencies such as self-esteem, self-efficacy, and group identification [69,70,71,72], emotional competencies such as empathy and alexithymia [10,23,73], social competencies such as relational skills, assertiveness, and conflict resolution [35,74], and cognitive competencies such as academic performance [16,75]. It should be noted that in our study, the improvement was especially relevant on the variables of self-efficacy, group identification, alexithymia, relational skills, and conflict resolution.

In the case of the other cognitive skills, such as planning and decision making, our hypothesis was not confirmed. These results are similar to those found for the Adventure of Life program, developed by EDEX in Spain [76] where there were no significant changes one year after the intervention. These findings suggest that the decision-making skill is complex and requires a series of previous processes and stages, as well as more time to develop.

There were no statistically significant differences in moral values either. Similar results are obtained with the Emotional Instructional Program for Personal Growth and Self-Realization (PIECAP), where only the older students connect more directly with the interests and concerns raised [77]. In addition, research by Berríos-Valenzuela andBuxarrais-Estrada [78] shows that adolescents would like to be valued for their level of intelligence, social skills, sensitivity, sympathy, responsibility, solidarity, personality, and friendliness. These aspects are related to the social factor, rather than to values that can be perceived as more abstract and distant from their daily lives, as occurs with this module.

This study has some limitations that should be taken into account when interpreting the results. Generalization of the results to the urban adolescent population should be viewed with caution. The program should be adapted to the characteristics of the urban adolescent population, and its effectiveness should be evaluated in subsequent studies. A longitudinal study should also be conducted to test the stability of long-term changes observed in the intervention group and find out whether the benefits are maintained, as previous work has shown [10]. Finally, another limitation could be the exclusive use of self-reports as assessment instruments, although previous studies highlight acceptable levels of reliability and validity of self-reports in adolescents [10].

Despite these limitations, it is important to note that this is the first study to include all the PYD dimensions, based on the model by Oliva et al. [3], with adolescent students in a rural setting. It contributes consistency to the effects and learning generated by the DPAR program that can be useful for its implementation in other schools. The results suggest the importance of fostering the relationship with young adults who reside in or are linked to the rural environment as a resource in the teaching-learning process and a facilitator of positive behaviors. Furthermore, the study shows that in a rural setting, the adolescent’s class is an effective group for improving social-emotional skills and positive assessment of the school. It opens up new avenues by addressing all the competencies in a holistic and integrated manner, leaving ample room for future research in other rural and urban contexts.

## 5. Conclusions

This study presents the DPAR program and evaluates its effects in a sample of early adolescents. The results support the initial hypotheses because significant improvements are observed in the intervention group, compared to the control group, in the variables of self-esteem, self-efficacy, group identification, empathy, relational skills, assertiveness, conflict resolution, alexithymia, and academic performance.

In summary, this study shows the effectiveness of the DPAR Program, demonstrating that it is a quality program and generates an adequate fit between the scenario and the environment [79]. In a rural setting, the school is the context where the greatest development among peers takes place; therefore, the DPAR Program is implemented in this context with the participation of members of the community. This program makes it possible to advance along the lines of version 3.0 of youth development programs, as Roth & Brooks-Gunn [11] proposed.

## Figures and Tables

**Table 1:** Relevant study variables based on PYD models.

		PYD Models	
Variables	Positive Adolescent Development Model [3]	Five Cs Model [6]	Developmental Assets [7]
Self-esteem, Self-efficacy, and Group Identification	Personal Area	Confidence and Competence	Positive Identity and Sense of belonging to the school
Relational Skills, Assertiveness, and Conflict Resolution	Social Area	Connection	Special competencies
Empathy and Alexithymia	Emotional Area	Caring	Special competencies
Planning and decision making	Cognitive Area	Competency	Special competencies
Moral Values	Moral Area	Character	Positive values
Academic Performance	Cognitive Area	Competency	Commitment to learning

**Table 2:** Sample characteristics in intervention and control groups.

Variables	Intervention Group (*n* = 83)	Control Group (*n* = 93)	*t/χ* ^2^ *(gl)*	*p*
Age Mean (SD)	12.83 (0.867)	12.95 (0.925)	0.847 (1, 174)	0.398
Sex			0.096 (1)	0.756
Boys	48.2%	50.5%		
Girls	51.8%	49.5%		
Grade level in Secondary Education			0.149 (1)	0.700
Grade 7	47%	44.1%		
Grade 8	53%	55.9%		
Academic Mark Mean (SD)	3.27 (1.21)	3.37 (1.32)	5.23 (1, 174)	0.601

**Table 3:** Structure, activities, and timing of the DPAR Program.

Module	Units	Activities		Time-Frame
		Classroom	Family	
MODULE I: Presentation of the program	Unit 1: DPAR program	1. What is the DPAR program? 2. Breaking the ice 3. Co-existence rules	1. Unwritten norms	September (two weeks)
MODULE II: Personal Competencies	Unit 2: Self-esteem Unit 3: Self-concept Unit 4: Self-efficacy Unit 5: Sense of belonging and identity	4. How I see myself 5. The range 6. Where I come from and where I’m going 7. This is my class; this is my group	2. Past photo vs. current photo 3. My parents’ life project	October—November (five weeks)
MODULE III: Emotional Competencies	Unit 6: Recognition of one’s emotions and those of others Unit 7: Emotional Regulation Unit 8: Empathy	8. Emotional alphabet 9. We read the same news together 10. The wax museum 11. The stoplight 12. My suitcase 13. I put myself in your place	4. Emotional mediator in the icebox 5. Cine at home 6. There’s space in my suitcase	November—December (five weeks)
MODULE IV: Social Competencies	Unit 9: Types of communication: passive, aggressive, and assertive Unit 10: Verbal and non-verbal communication Unit 11: Relational skills Unit 12: Conflict resolution	14. We do some theater 15. The messenger ball 16. The history of the barn 17. You deserve it 18. How to give hugs? 19. Knowing how to say NO 20. Let’s untie the knot	7. Words take care of us 8. My gift: gratitude	January—February (five weeks)
MODULE V: Cognitive competencies	Unit 13: Capacity for analysis Unit 14: Creativity Unit 15: Capacity to plan and revise Unit 16: Capacity to make decisions	21. Improved version of the school 22. Creative brain 23. My time is golden 24. Intelligent decisions	9. Improved version of my house	March—April (four weeks)
MODULE VI: Moral Competencies	Unit 17: Social commitment and responsibility Unit 18: Prosocial attitudes Unit 19: Equality and respect for diversity	25. Volunteering 26. Support group 27. I am a reporter (I) 28. I am a reporter (II)	10. My family’s values 11. Did you know that…	May (four weeks)
MODULE VII: Closing the program	Unit 20: Saying good-bye to the program	29. We learned together 30. Preparation for Open-doors day		June (two weeks)

**Table 4:** Descriptive statistics and repeated-measures ANOVA for PYD variables.

Variables			*M* (*SD*)			*F^b^ (p)*			$n_{p}^{2}$
	Group	Pre-Test	*t^a^*	*p*	Post-Test	Time Effect	Group Effect	Interaction Effect	
Self-esteem	Intervention	2.87 (0.55)	1.56	120	3.30 (0.57)	29.13 ***	0.032	6.79 **	0.04
	Control	2.98 (0.41)			3.15 (0.45)				
Self-efficacy	Intervention	2.76 (0.54)	0.48	0.140	3.22 (0.53)	20.47 ***	0.862	8.71 **	0.05
	Control	2.89 (0.58)			2.98 (0.66)				
Group identification	Intervention	7.25 (1.43)	0.515	0.607	8.71 (1.07)	13.88 ***	3.14	5.48 *	0.03
	Control	7.36 (1.44)			8.13 (1.46)				
Empathy	Intervention	3.53 (0.83)	1.12	0.264	4.08 (0.56)	25.60 ***	0.372	5.30 *	0.03
	Control	3.65 (0.62)			3.87 (0.60)				
Alexithymia	Intervention	3.38 (0.89)	−2.27	0.024	2.59 (0.91)	37.23 ***	0.958	9.09 **	0.05
	Control	3.11 (0.66)			2.82 (0.73)				
Relational skills	Intervention	4.93 (1.26)	1.62	0.106	5.50 (1.47)	45.54 ***	5.27 *	7.43 **	0.06
	Control	3.99 (1.14)			4.46 (1.06)				
Assertiveness	Intervention	5.35 (0.99)	0.159	0.874	6.16 (0.74)	33.70 ***	10.05 **	12.14 **	0.06
	Control	5.33 (1.01)			5.53 (0.88)				
Conflict resolution	Intervention	3.80 (1.36)	2.95	0.004	4.99 (1.50)	23.70 ***	0.26	12.78 ***	0.07
	Control	4.36 (1.26)			4.54 (1.24)				
Planning and decision making	Intervention	4.76 (0.91)	3.76	0.001	5.12 (1.16)	4.44 *	11.57 ***	1.67	0.01
	Control	5.30 (0.95)			5.42 (1.13)				
Moral values	Intervention	4.76 (0.85)	0.51	0.133	5.08 (0.85)	11.53 ***	1.57	0.102	0.001
	Control	4.96 (0.84)			5.17 (0.86)				
Academic performance	Intervention	3.27 (1.21)	0.523	0.601	3.81 (0.803)	48.64 ***	0.568	25.64 ***	0.128
	Control	3.37 (1.32)			3.45 (1.20)				

*Note. t^a^*: t-test comparing pre-test means in the intervention and control groups *F^b^*: F values for main and interaction effects in ANOVA * p< 0.05; ** *p*< 0.01; *** *p*< 0.001.

**Table 5:** Simple effects analysis.

Variables					95% CI		
	Group	Mean dif. (I-J)	Std. Error	Sig. ^a^	LL	UL	Cohen’s *d*
Self-esteem	Intervention	0.419	0.073	0.001	0.274	0.564	0.79
	Control	0.146	0.075	0.052	−0.002	0.294	0.32
Self-efficacy	Intervention	0.459	0.088	<0.001	0.285	0.633	0.84
	Control	0.097	0.086	0.260	−0.072	0.265	0.14
Group identification	Intervention	1.46	0.211	0.001	1.04	1.87	1.15
	Control	0.771	0.207	<0.001	0.362	1.18	0.53
Empathy	Intervention	0.554	0.109	<0.001	0.339	0.769	0.79
	Control	0.207	0.104	0.058	0.002	0.413	0.36
Alexithymia	Intervention	−0.817	0.132	0.001	−1.07	−0.555	0.89
	Control	−0.277	0.121	0.023	−0.515	−0.038	0.41
Relational skills	Intervention	0.059	0.231	0.001	1.11	2.03	0.94
	Control	0.846	0.246	0.798	0.359	1.33	0.14
Assertiveness	Intervention	0.839	0.130	0.001	0.583	1.09	0.75
	Control	0.210	0.126	0.096	−0.038	0.458	0.21
Conflict resolution	Intervention	1.20	0.205	0.001	0.797	1.60	0.82
	Control	0.184	0.197	0.353	−0.206	0.574	0.12
Academic performance	Intervention	0.542	0.065	<0.001	0.413	0.480	0.52
	Control	0.086	0.062	0.166	−0.036	0.208	0.06

Note. CI: Confidence Interval; LL = Lower Limit CI; UL: Upper Limit CI ^a^. Adjustment for multiple comparisons: Bonferroni.
